# DNA and RNA Methylation in Rheumatoid Arthritis—A Narrative Review

**DOI:** 10.3390/epigenomes9010002

**Published:** 2025-01-08

**Authors:** Kajetan Kiełbowski, Estera Bakinowska, Anna Gorący-Rosik, Karolina Figiel, Roksana Judek, Jakub Rosik, Paweł Dec, Andrzej Modrzejewski, Andrzej Pawlik

**Affiliations:** 1 Department of Physiology, Pomeranian Medical University, 70-111 Szczecin, Poland; kajetan.kielbowski@onet.pl (K.K.); esterabakinowska@gmail.com (E.B.); kfigiel344@gmail.com (K.F.); judek.roksana@gmail.com (R.J.); jakubrosikjr@gmail.com (J.R.); 2 Department of Clinical and Molecular Biochemistry, Pomeranian Medical University, 70-111 Szczecin, Poland; ania.goracy@gmail.com; 3 Department of Plastic and Reconstructive Surgery, 109 Military Hospital, 71-422 Szczecin, Poland; 4 Clinical Department of General Surgery, Pomeranian Medical University in Szczecin, Piotra Skargi 9-11, 70-965 Szczecin, Poland; amodrzejewski@interia.pl

**Keywords:** rheumatoid arthritis, epigenetics, DNA methylation, RNA methylation

## Abstract

Rheumatoid arthritis (RA) is a progressive autoimmune disease leading to structural and functional joint damage and, eventually, to physical disability. The pathogenesis of the disease is highly complex and involves interactions between fibroblast-like synoviocytes (FLSs) and immune cells, which stimulate the secretion of pro-inflammatory factors, leading to chronic inflammation. In recent years, studies have demonstrated the importance of epigenetics in RA. Specifically, epigenetic alterations have been suggested to serve as diagnostic and treatment biomarkers, while epigenetic mechanisms are thought to be involved in the pathogenesis of RA. Epigenetic regulators coordinate gene expression, and in the case of inflammatory diseases, they regulate the expression of a broad range of inflammatory molecules. In this review, we discuss current evidence on the involvement of DNA and RNA methylation in RA.

## 1. Introduction

Rheumatoid arthritis (RA) is one of the most common autoimmune diseases. This chronic, progressive systemic disease affects up to 0.25–2% of the global population [1,2,3]. This inflammatory connective tissue disease is characterized by non-specific synovitis with excessive synovial proliferation, cartilage degradation, and the destruction of joints and periarticular areas, leading to functional impairment, severe disability, or even premature death. Symptoms most often reported by patients with RA include symmetric inflammation of small joints with tenderness and morning stiffness. The inflammation starts at smaller joints [3].

RA prevalence depends on a variety of factors, including age, geographical location, and gender, with a higher prevalence in women [4,5]. Genetic and genomic studies, especially large-scale genome-wide association studies (GWASs), have enabled the detection of many risk genes. Some are associated with proinflammatory cytokines, signal transducers, or transcriptional activators [6]. Other polymorphisms, like the one affecting a critical sequence of five amino acids of the HLA-DRβ chain, are one of the strongest risk factors of RA [3]. Nevertheless, amongst monozygotic twins, the disease concordance rate for RA is ~15% [7]. Despite impressive progress in genetic methodology and several decades of extensive research, the etiopathogenesis of lots of RA cases is still undiscovered. A large part of recently accumulated data show the potential influence of epigenetics mechanisms in RA pathology [8]. Moreover, despite the availability of modern targeted and biological DMARDs, a proportion of patients fail to respond adequately, which is known as difficult-to-treat RA [9]. Furthermore, delayed or suboptimal treatment can result in joint damage and functional decline, highlighting the importance of early intervention for better long-term outcomes [3,10]. Additionally, RA patients experience a significantly increased mortality risk compared to the general population [11]. Understanding the role of epigenetic background in the complex interplay between genetic and environmental factors might overcome these obstacles in treatment and lead to novel efficacious therapies for RA. This promising avenue of research offers hope for the improvement of the lives of countless patients.

Joint homeostasis disturbance in RA is associated with cells forming the intimal lining layer of synovium, fibroblast-like synoviocytes (FLSs). They produce synovial fluid and control the composition of the extracellular matrix (ECM) [12]. RA-FLSs play a central role in disease pathogenesis by becoming activated in response to inflammatory signals and releasing inflammatory mediators, including cytokines (IL-1β, IL-6), together with matrix metalloproteinases (MMPs) that damage extracellular matrix, among others [13,14]. Differences between FLS phenotype in the healthy general population and RA patients might be associated with epigenetic imprint affecting cells’ ability to avoid apoptosis and increase proteasomal activity [8,15,16]. Data from epigenomic profiling of autoimmune diseases seems intriguing and promising. Epigenomic profiling techniques include DNA methylation sequencing, histone modification ChIP-seq, and RNA methylation sequencing. They were pivotal in uncovering the RA molecular mechanisms described in this article. Due to data complexity, these analyses require the application of various bioinformatic methods [17,18]. DNA methylation sequencing reveals changes, mainly in gene promoters, which help to identify genes aberrantly regulated in RA patients [19,20,21]. Histone modification ChIP-seq allows for the examination of chromatin states, highlighting regions of active transcription or repression [22]. Additionally, RNA methylation sequencing, especially for m6A modifications, uncovers the influence of RNA processing and translation alterations on the dysregulated immune response [23]. Global methylation analysis was used to distinguish between RA patients and healthy controls [24]. Moreover, early changes in DNA methylation of FLSs are detectable with the aforementioned methods before clinical diagnosis [25]. Results obtained with these novel techniques might also be transferred into treatment response biomarkers [26].

**Figure 1:**
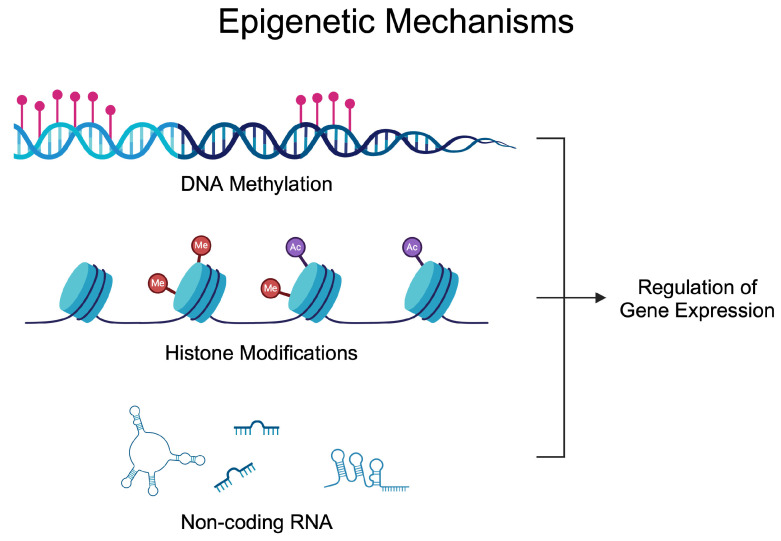
Epigenetic mechanisms regulating gene expression. Over the years, studies have demonstrated dysregulated epigenetic processes in rheumatoid arthritis that contribute to the progression of the disease and could be utilized in the diagnosis process. Created in BioRender. Kiełbowski, K. (2024) BioRender.com/l26w541 (accessed on 15 May 2024).

Epigenetics investigates alterations in gene activity occurring without a change in the nucleotide sequence [27]. The epigenetic regulation of gene expression depends on the balance of enzymes modifying DNA, RNA, and histones. These modifications enable the precise adjustment of gene expression in response to internal and external signals [28,29]. Epigenetic alterations have been known for many years to participate in the development of many diseases, thus suggesting their potential targeting [30,31,32,33,34]. The most common epigenetic mechanisms of gene expression are DNA methylation, RNA methylation, histone modification, chromatin remodeling, and non-coding RNAs (ncRNAs) [35]. A significant number of epigenetic-based agents have been approved for the treatment of various types of diseases, mostly developed to treat cancer, nonetheless. Azacitidine (DNA methylation inhibitor) [36], vorinostat (histone deacetylase inhibitor) [37], or givinostat (histone deacetylase inhibitor) [38]. Nowadays, these drugs are being repurposed, as novel evidence demonstrates the activity of those agents in inflammatory conditions [39]. Figure 1 demonstrates epigenetic mechanisms that affect gene expression. Briefly, DNA methylation can alter the accessibility of transcription factors. Similarly, histone modifications change chromatin composition, thus allowing for transcription to occur at specific regions, while ncRNA utilizes various methods to mediate gene expression. Micro RNA (miRNA) can bind to the complementary mRNA and suppress translation. Long non-coding RNA (lncRNA) and circular RNA (circRNA) can act as sponges and interact with miRNAs to reverse their inhibitory effects.

Methylation assays allow researchers to assess various methylation sides to identify disease biomarkers and new molecular targets for epigenetic therapy. Moreover, progress in the understanding of RNA biology has contributed to novel advances in the diagnosis and therapy of numerous diseases. Epigenetic therapies, while promising, are associated with several adverse side effects, particularly cytotoxicity and immunosuppression. These approaches might alter epigenetic processes in every cell in the organism, leading to hematological, cardiovascular, or gastrointestinal side effects [40,41]. DNA methyltransferase (DNMT) inhibitors, such as azacitidine and decitabine, used for myelodysplastic syndromes and leukemias, can cause significant bone marrow suppression. This leads to hematologic toxicities like neutropenia, anemia, and thrombocytopenia, consequently increasing risks of infections, fatigue, and bleeding, respectively [42]. Histone deacetylase (HDAC) inhibitors that are registered in the treatment of malignancies can cause side effects such as fatigue, nausea, vomiting, and diarrhea, significantly impacting patients’ quality of life [43,44].

Epigenetics and methylation were explored in the context of autoimmune diseases for decades. Studies published in the 1980s and 1990s demonstrated the importance of DNA methylation in the behavior of T cells and altered methylome profiles in patients with autoimmune diseases [45,46]. More recent investigations revealed more precise mechanisms linking RA with DNA and RNA methylation, together with other epigenetic processes.

The aim of this review is to summarize the role of DNA and RNA methylation in the pathogenesis of RA and discuss the potential application of this knowledge clinically. Analyses of the methylome could provide novel opportunities for RA diagnosis and treatment biomarkers, thus improving treatment outcomes and quality of life. This paper aims to address gaps in the existing literature and suggest clinically beneficial approaches.

## 2. The Role of Epigenetics in Health and Disease

DNA and RNA methylation are pivotal epigenetic modifications that exert considerable influence over the regulation of gene expression and genome stability. This process typically involves the addition of a methyl group to the fifth carbon position of cytosine residues [47], with this modification occurring predominantly at cytosine-phosphate-guanosine (CpG) dinucleotides.

The process of DNA methylation can suppress gene expression by modifying the chromatin structure, making it less accessible to transcription factors [48]. DNA methylation plays a particularly important role in the regulation of T cells, which is crucial in the context of autoimmune diseases. Changes in DNA methylation can result from environmental factors, genetic variants, drugs, and miRNA [47].

Enzymes are crucial for epigenetic mechanisms. DNA methylation is catalyzed by DNMTs, which utilize S-adenosyl-L-methionine (SAM) as the methyl group donor [49]. The resulting 5-methylcytosine (5mC) functions as a pivotal epigenetic marker, influencing a vast range of biological processes, including transcriptional repression, X chromosome inactivation, genomic imprinting, and the maintenance of chromosomal stability [49]. It should be noted that DNA methylation is not a static mark; rather, it can be dynamically regulated by the addition and removal of methyl groups.

DNMT1 plays a pivotal role in maintaining DNA methylation during cell division, thereby ensuring the faithful transmission of the epigenetic mark to daughter cells. In contrast, DNMT3A and DNMT3B are involved in de novo methylation, which involves the addition of methyl groups to previously unmethylated DNA regions [29,50]. This process is of particular importance during embryonic development and in response to environmental cues. TET enzymes (which catalyze the sequential oxidation of 5-methylcytosine (5mC) in DNA) act to counterbalance the activity of DNMTs by oxidizing 5-methylcytosine, thereby facilitating its removal. This enables the dynamic regulation of DNA methylation and contributes to processes such as gene activation and cellular reprogramming [50]. Environmental factors such as diet and stress also induce epigenetic alterations that contribute to chronic inflammation and aging-related diseases [51]. DNA methylation changes have been identified in autoimmune diseases such as RA, contributing to disease progression and treatment response [51].

DNA methylation plays a crucial role in the regulation of the immune system. For example, regulatory T cells (Tregs) play a significant role in autoimmune diseases. Specifically, imbalance between Tregs and pro-inflammatory Th17 cells arises in autoimmunity. Treg cells express a Foxp3 transcription factor. In chronic inflammation, the expression of Foxp3 is reduced, which is associated with autoimmunity [52]. A study by Tseng et al. [53] demonstrated that tumor necrosis factor receptor 2 (TNFR2) reduces the DNA methylation of Foxp3 promoter, which allows for maintaining the expression of Foxp3 in Treg cells. Moreover, the authors revealed that a deficiency of TNFR2 resulted in a more severe course of arthritis with a lower number of Treg cells [53]. Furthermore, DNA methylation affects macrophage polarization. Despite T cells, macrophages are significantly implicated in the pathogenesis of inflammatory diseases. The M1 pro-inflammatory cells are major sources of tumor necrosis factor α (TNF-α), a cytokine driving the progression of RA. Agents that modulate DNA methylation processes affect macrophage polarization [54].

DNA methyltransferases and histone deacetylases represent promising targets for novel therapeutic interventions, providing new treatment options for cancers and autoimmune disorders. Targeting these enzymes can modulate epigenetic states, potentially reversing aberrant gene expression patterns associated with disease progression and improving clinical outcomes [55]. Epigenetic changes play a crucial role in the pathogenesis of autoimmune diseases by modulating immune responses and promoting inflammatory states. These alterations can affect the expression of key immune-related genes, leading to the dysregulation of the immune system and contributing to the development and exacerbation of autoimmune conditions [47]. Furthermore, it enables the investigation of how lifestyle factors, such as diet and stress, can lead to changes in gene expression associated with various diseases [56]. It has been demonstrated that lifelong environmental exposure to nutritional, endocrine, and chemical disruptions can alter gene expression through epigenetic modifications, such as DNA methylation, histone modification, and microRNA involvement, thereby influencing the phenotypic differentiation of cells [57]. Consisting of 18 members, the histone deacetylase (HDAC) family serves as a group of epigenetic regulators crucial for the chromatin-mediated control of gene expression [58]. HDAC inhibitors (HDACis) are anti-cancer agents undergoing oncology and rheumatology indication trials due to well-documented anti-inflammatory activities [58,59]. HDACi treatment might improve the outcome of autoimmune and autoinflammatory-like inflammatory bowel disease (IBD) or RA through the induction of FLS growth arrest, anti-angiogenic properties, or suppression of pro-inflammatory cytokines like IL-6 and TNF-α [59,60,61,62]. Particularly, the targeting of HDAC1, 2, and 6 and inhibitors such as trichostatin A, suberoyl bis-hydroxamic acid, nicotinamide, and suberoyl anilide hydroxamic acid were found to influence synovial inflammation [60,62,63]. Sirtuins (SIRTs) represent a group of nicotinamide adenine dinucleotide (NAD)-dependent HDACs. Seven members of SIRTs were identified in mammals [64], and recent studies uncovered their crucial role in regulating physiological and pathological processes. In RA, these enzymes regulate angiogenesis [65] and inflammation [66], thus profoundly affecting the progression of RA. Poniewierska-Baran and colleagues [67] nicely reviewed the involvement of SIRTs in RA.

Interestingly, epigenetic mechanisms also affect each other, thus creating a broad regulatory network. For instance, ncRNA can affect the expression of DNMTs, therefore changing not only gene expression but also methylation profile [68]. Unlike genetic mutations, epigenetic modifications are reversible, which creates potential opportunities for therapeutic interventions that can restore normal patterns of gene expression [69]. Understanding their roles in disease can lead to novel therapeutic strategies and biomarkers for the early detection and treatment of various conditions [70].

## 3. DNA Methylation and Rheumatoid Arthritis

### 3.1. DNA Methylation in the Pathogenesis of Rheumatoid Arthritis and Therapeutic Implications

DNA methylation is an important epigenetic mechanism that regulates gene expression. Over the years, researchers have demonstrated alterations in the methylation profile in patients with inflammatory diseases. Taking into consideration the influence of methylation on gene expression, altered methylome could be involved in the pathogenesis of different conditions.

Researchers have been studying DNA methylation in RA patients and models for several decades. These early studies showed a reduced global methylation profile in patients with RA [71]. Moreover, studies published over a decade ago also showed different expressions of enzymes catalyzing the DNA methylation process. Compared to controls, mRNA expression of DNMT1 was increased in the RA cohort [72]. However, studies have also shown that the expression of these enzymes changes depending on the inflammatory conditions. Nakano et al. demonstrated that pro-inflammatory cytokines reduced the expression of DNMT1 and DNMT3a in FLSs [73]. Furthermore, increased expression of demethylating enzymes was found in some populations of immune cells in an RA population [74]. Thus, early studies demonstrated that the state of RA alters the DNA methylation profile and the expression of enzymes catalyzing these processes. Subsequent studies began investigating the involvement of these observations in the pathogenesis of RA, together with their potential clinical utility. A bibliometric analysis demonstrated a positive trend in the number of papers examining DNA methylation in RA, indicating an increasing number of discoveries [75].

Studies have suggested that a potential relationship between methylation profile and inflammatory markers exists in RA patients. Significant positive and negative correlations between the methylation levels of the respective chemokine CXCR5 and HIPK3 with CRP were observed [76,77]. Interestingly, in a recently published paper by Wielscher et al., researchers comprehensively studied links between CRP and DNA methylation and identified 1511 CpG sites associated with CRP. Using Mendelian Randomization triangulation analysis, the authors investigated the causal links between CpG methylation sites and CRP. The results suggested that CRP affects the methylated CpGs [78]. Therefore, chronic inflammation associated with elevated CRP levels could cause alterations in DNA methylation, which would further affect inflammatory mechanisms. Based on the discussed studies, patients with RA have a reduced methylation profile and an altered expression of enzymes catalyzing methylation reactions, while chronic inflammation associated with the disease could contribute to the changes in methylome. A question arises as to whether the modification of the methylation status could affect pathologic processes in RA. Gaur et al. [79] examined this issue and supplied RA-FLSs with L-methionine and betaine, which would be expected to enhance remethylation. The former agent significantly enhanced the DNA methylation of these cells. Additionally, cells treated with L-methionine demonstrated an altered expression profile of miRNAs. Specifically, stimulation with this methyl donor upregulated let7f, miR-9, miR-29, and miR-203. Mechanistically, these molecules could inhibit the remethylation process, and such finding was observed regarding miR-29. DNA methylation was inversely correlated with miR-29 expression, and further analyses confirmed that miR-29 targeted and regulated the expression of DNMT3A. Interestingly, even though betaine was unsuccessful in stimulating DNA methylation, it showed other beneficial effects, such as a decreased expression of MMP-1 and a suppressed migration of RA cells [79]. Thus, this study revealed several important findings: RA-FLSs can be treated with agents that increase DNA methylation. Secondly, these agents could improve the behavior of rheumatic cells and reduce their invasiveness. Furthermore, RA-FLSs possess resistance mechanisms that can suppress the remethylation process. Importantly, it is widely known that miRNAs are involved in a broad interaction network and frequently target multiple mRNAs, while they are also regulated by numerous other molecules, such as lncRNAs and circRNAs. Regarding miR-29-3p, it was recently found that this molecule regulates the expression of mouse TNF receptor 1 [80], which suggests that modifying miR-29-3p expression might influence inflammatory processes in several mechanisms. These findings show that epigenetic mechanisms are tightly linked with each other, and regulating their functionality can induce thorough changes in signaling and cell responses. Moreover, more complex and thorough studies are required to understand the links, regulatory networks, and clinical translation of epigenetic mechanisms. With the growing popularity of epigenome-wide association studies (EWASs) that analyze thousands of CpG sites [81], more extensive knowledge and understanding of the role of DNA methylation in RA should be expected. A combination of genetic and epigenetic studies further helps us to understand the complex mechanisms associated with RA. Julia and colleagues [82] found 64 differently methylated CpG sites between RA patients and controls. A replication analysis confirmed 10 sites associated with RA. Moreover, a combination of CpG sites and single nucleotide polymorphisms (SNPs) confirmed three variants linked to RA risks [82]. The combination of genetics and epigenetics is expected to introduce personalized treatment in patients with RA. Pharmacogenetics aims to identify genetic variants associated with treatment resistance. Researchers look for genetic variants associated with the response or differentially expressed genes [5,83]. Combining this knowledge with epigenetics could further increase our understanding of the disease’s pathogenesis and suggest potential therapeutic strategies. Ha and colleagues [84] suggested that differentially expressed genes could result from differentially methylated regions. Researchers have found that these mechanisms apply to genes associated with CD4+ T cells’ functionality. A wider understanding of these regulatory networks could create advanced prediction models that would guide treatment methods.

### 3.2. DNA Methylation and Biomarker Identification

Monitoring DNA methylation could provide clinically useful information. For instance, methylation could be used in the diagnosis process. Furthermore, it could be used to evaluate treatment response.

Firstly, a methylation analysis of a single gene was found to discriminate RA patients from healthy control. In a study by Gravand et al., researchers found that levels of *CDKN2A* promoter methylation in RA patients are significantly lower compared to controls. The ROC curve analysis demonstrated a moderate diagnostic power (area under curve, AUC, of 0.705) [85]. A higher diagnostic potential was observed regarding the methylation of HIPK3 (AUC 0.821). Importantly, the implementation of HIPK3 was also suggested to significantly improve diagnostic power in RF-/CCP- patients [77]. Therefore, monitoring DNA methylation could help in diagnosing seronegative patients. Additionally, epigenetic data can help in discriminating RA patients from those with other forms of arthritis. Specifically, lower methylation levels of the *TNF* gene were suggested to predict RA diagnosis among patients with psoriatic arthritis, reactive arthritis, and undifferentiated arthritis [86]. In another study, the authors demonstrated that CG sites have significant correlations with clinical parameters, such as the number of tender and swollen joints, as well as RF and CCP levels [87]. Moreover, epigenetic data could be used to evaluate the introduction of adequate treatment methods, as a different methylation profile of FLSs based on joint of origin was found [88]. Combining the data about DNA methylation and the expression of inflammatory factors could indicate different treatment agents for a disease affecting particular joints.

**Figure 2:**
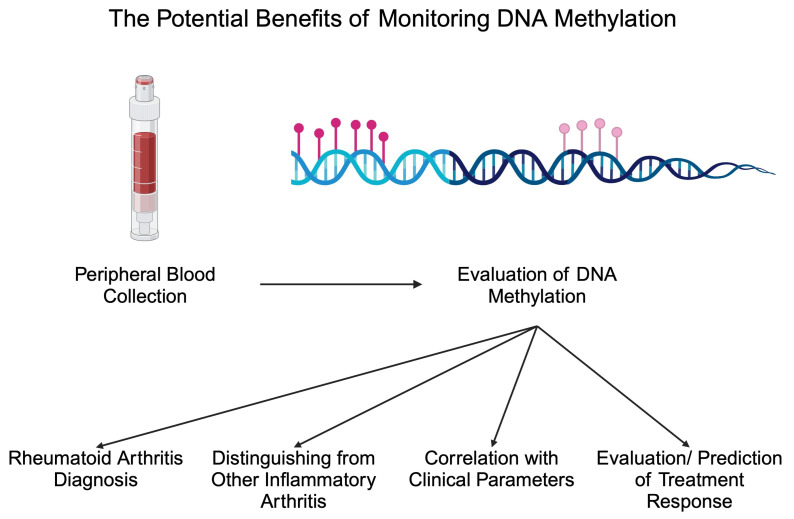
Potential benefits associated with monitoring DNA methylation in patients with rheumatoid arthritis. Created in BioRender. Kiełbowski, K. (2024) BioRender.com/j38i306 (accessed on 15 May 2024).

Apart from diagnostic biomarkers, markers of treatment response are greatly needed in patients with inflammatory disorders. The monitoring of such markers could introduce more personalized treatment methods or potentially stop treatment in patients with inactive disease. Methotrexate (MTX) is a crucial conventional synthetic DMARD (csDMARD) that is being given to the majority of RA patients. Recently, researchers suggested that the monitoring of DNA methylation could be applied to monitor MTX response. In an analysis by de Andres et al., the authors observed that one month of MTX treatment increased DNA methylation in peripheral blood cells. Specifically, the authors noted increased methylation in T cells, B cells, and monocytes [74]. In addition, a different methylation profile has been observed between responders and non-responders to MTX, thus highlighting the possible adjuvant role in response evaluation [89]. In an important study by Nair and collaborators, the authors analyzed methylation 4 weeks after the beginning of treatment with MTX and found that these data could be used to predict swollen joint counts and CRP levels 6 months after MTX initiation [90]. Due to the limited response rate to MTX, the identification of biomarkers that could discriminate non-responders at an early stage after the beginning of treatment would allow for rapidly changing treatment and reducing the exposure to MTX, thus reducing the risk of adverse events. Interestingly, a recent study by Ravaei et al. [91] suggests the potential involvement of long interspersed nucleotide element-1 (LINE-1) methylation in predicting MTX response. LINE-1 creates repetitive sequences in the genome, and it is estimated that it comprises 17% of the human genome. LINE-1 element is a retrotransposon that uses RNA to insert new “copy-and-paste” elements in the genome [92]. The inhibition of LINE-1 mobility was previously suggested to reduce invasive features and responses of synovial cells [93], thus suggesting the possibility of its involvement in the pathophysiology of RA. Regarding DNA methylation, Ravaei and colleagues showed that LINE-1 methylation is significantly different between moderate and good responders to MTX in the respective cohorts of RF-, RF+, ACPA-, and ACPA+ patients [91]. Based on the recent analysis of the whole-genome DNA methylation, this epigenetic marker can be implemented in predicting the response to leflunomide, a different member of the csDMARD therapeutic group. Specifically, a combination of seven differently methylated positions with age at diagnosis and lymphocytes count represents a model combining both DNA methylation and clinical data, which shows a promising predictive power [94]. Researchers also began investigating the potential monitoring of DNA methylation to detect responses to bDMARDs, such as adalimumab. Recently, Hageman showed that the methylation of 20 CpG sites was associated with a response to adalimumab, which was independent of smoking or concomitant MTX administration [26]. Thus, monitoring DNA methylation could provide numerous clinically beneficial data regarding RA patients in the future (Figure 2). Table 1 summarizes selected studies investigating the altered methylome in RA.

## 4. RNA Methylation and Rheumatoid Arthritis

### 4.1. RNA Methylation in the Pathogenesis of Rheumatoid Arthritis and Therapeutic Implications

Different epigenetic modifiers have been reported to influence the course of autoimmune diseases such as RA. Scientists have already identified several genes associated with RNA modifications. These genes encode enzymes involved in alternating RNA molecules. The expression of these genes may be affected by changes in their RNA methylation levels [95]. N6-methyladenosine (m6A), N1-methyladenosine (m1A), and cytosine-5 methylation (m5C) are some of the RNA modification examples that might contribute to the development of RA [23].

RNA methylation plays a critical yet distinct role in regulating gene expression compared to DNA methylation. DNA methylation acts as a long-term epigenetic modification by affecting gene silencing. It alters chromatin structure and the binding of the transcription factor to the promoter region and restricts their accessibility to DNA-binding sites [96]. RNA methylation operates post-transcription and modulates RNA stability, splicing, translation, and degradation. The most prevalent modification, m6A, modifies the mRNA fate, eventually coordinating protein formation [97]. DNA methylation in RA leads to more stable, heritable changes. RNA methylation is reversible and provides more dynamic, transient changes for regulating gene expression in response to immune signaling [98]. RNA and DNA methylation are one of the underlying mechanisms of disease pathogenesis. DNA methylation typically changes the expression of key immune-related genes, particularly at CpG islands in gene promoters [99]. RNA methylation plays a role in regulating RNA processing and translation, which influences the expression of cytokine mRNAs and immune modulator genes such as *ADAMDEC1* and *IGHM* more context-dependently [23]. Therefore, both modifications are implicated in RA development, but RNA methylation probably offers a more flexible and rapidly activating way of regulation than abiding changes in DNA methylation.

Methylation at the N6 site of the adenine base (m6A) is the most prevalent posttranscription modification of RNA. Aberrant m6A modifications conducted by m6A methylation regulators have already been associated with aging and various diseases like heart failure, nonalcoholic fatty liver diseases, cancers, neurodevelopment, and autoimmune diseases, including RA [100,101,102]. Three types of enzymes catalyzing m6A modifications are distinguished. The members of the first group are methyltransferases known as “writers” and include methyltransferase-like 3 (METTL3), methyltransferase-like14 (METTL14), and their cofactors—Wilms tumor 1-associated protein (WTAP) and RNA-binding motif protein 15 (RBM15/15B). m6A demethylases are called “erasers”. This cohort consists of fat mass and obesity-associated protein (FTO) and ALKB family member 5 (ALKBH5). The last group contains “readers” like YTHDF1/2/3, YTHDC1/2, hnRNPC, hnRNPG and hnRNPA2B1, and IGF2BPs [103]. The “writers” and “erasers” participate in the dynamic and reversible processes of m6A methylation. The “readers” recognize m6A modifications and take part in the recruitment of downstream functional complexes. m6A modifications have been reported to play an important role in RA development. These modifications, via affecting m6A “writers”, “erasers”, and “readers”, impact multiple biological processes like RNA transcription, splicing, maturation, degradation, metabolism, localization, and translation [104,105].

m^6^A methylation is catalyzed by the methyltransferase complex (MTC). This complex consists of two components: METTL3 and METTL14. The MTTTL3-METTL14 complex takes part in the deposition of m^6^A on nuclear RNA [95]. Cross-links among m6A methylation regulators have been reported to be involved in developing various autoimmune diseases. Luo et al. showed lower expressions of METTL14, ALKBH5, and YTHDF2 than healthy controls in SLE patients [106]. Another study revealed that METTL3, METTL14, YTHDF1, and WTAP were involved in regulating IBD-related genes [107]. A study by Xie et al. demonstrated a high level of the TNF-α-induced directional migration of ankylosing spondylitis mesenchymal stem cells compared to mesenchymal stem cells from healthy controls by increasing ELMO1 expression. It was caused by the METTL14-mediated m^6^A modification of *ELMO1* [108]. Song et al. indicated that METTL3 may play a part in the occurrence of Graves’ disease by inducing m6A methylation modification of the suppressor of cytokine signaling family members [109].

METTL3 is a critical enzyme in m6A methylation modification. It regulates inflammation and autoimmune balance. Wang et al. [110] hypothesized that METTL3 might affect the course of RA by coordinating macrophage-mediated inflammation. It is indicated that METTL3 can prevent macrophage proliferation and the production of inflammatory cytokines, such as IL-6 and TNF-*α*, which are considered pathogenic in RA [111]. Su et al. reported elevated m6A methylation levels and METTL3 expression in synovial tissues and FLSs of RA patients, meaning that these processes may be related to the synovitis of RA. METTL3 and METTL14 levels were up-regulated in RA synovial tissues, and, additionally, *WTAP*, *FTO*, and *ALKBH5* were significantly elevated in synovial tissues and also FLSs [112]. In contrast, the level of METTL14 in PBMCs from RA patients was reduced compared to patients in remission, according to Tang et al. [113]. Fan et al. determined that ALKBH5 expression was higher in RA. The hypoxia-induced higher expression of ALKBH5 increased inflammation and the migration of RA-FLSs by affecting CH25H mRNA stability. They established that the HIF1α/2α-ALKBH5-CH25H pathway may be important for FLS activity and inflammation while providing a new approach to RA treatment based on targeting this pathway [114]. Moreover, decreased m5C modification, which has been marked in the synovial tissue of RA patients, is associated with elevated inflammation and, therefore, with RA severity [23]. Zhou et al. showed that the dysregulation of these RNA modification-related genes, such as *DNMT2*, *NSUN2*, and *TRDMT1*, as well as the m5C modification, may lead to the development of RA [115]. In general, all of the above findings point out that the dysregulation of RNA modification-related genes and their modifications may play a crucial role in the pathogenesis of RA.

FLSs play a critical role in the pathogenesis of RA by participating in pro-inflammatory reactions like metalloproteinases and cytokines production, osteoclast activation, and immune cell recruitment [116]. FLSs’ abnormal proliferation and their cartilage invasion are crucial for RA development. Therefore, discovering new drugs that suppress the pathological functions of RA-FLSs remains a potential strategy for new RA treatment. Chen et al. showed that artemisitene (ATT) could inhibit proliferation, migration, and invasion and promote apoptosis in RA-FLSs [117]. Shi et al. [118], in their study, reported that ATT, by inhibiting METTL3, modulates FLSs proliferation, apoptosis, migration, and invasion. Moreover, they demonstrated the significance of m6A RNA methylation connected with the antirheumatic activity of ATT by observing the suppressive impact of ATT on the METTL3-mediated m6A alteration of *ICAM2* mRNA in RA-FLSs. In addition, considering the anti-inflammatory properties of ATT, these studies may serve as a basis for the clinical usage of ATT in RA therapy [118,119]. To the best of our knowledge, the clinical effect of this drug in RA patients has not been examined yet. Jiang et al. identified differential m6A methylation and mRNA expression after stimulation with TNF-α in MH7A cells (RA-FLSs). The researchers presented 206 differentially expressed m6A methylation genes and 1207 differentially expressed mRNAs. These potential genes, through m6A methylation, are suspected to contribute to the development of RA [120]. Table 2 briefly summarizes selected RNA modifications and their probable involvement in RA.

As many new epigenetic modifiers have been developed in the last couple of years, it is predicted that innovative RNA-targeted therapies will bring high efficiency and specificity in the treatment of autoimmune diseases. RNA methylation-related enzymes and their targets may become new diagnostic tools or therapeutic targets. Accordingly, targeting, for instance, m6A regulators may be an achievable strategy for RA therapy. Chronic and prolonged treatment with common antirheumatic drugs constantly causes unpleasant side effects. Thus, it is crucial to continue developing novel antirheumatic treatments. Nonetheless, further studies are needed to establish the regulatory mechanisms of RNA modification in RA. Additional studies are required to develop new targets used for future clinical treatments.

### 4.2. RNA Methylation and Biomarker Identification

Wang et al. determined the expression of m6A methylation-associated genes (*METTL3*, *FTO*, *ALKBH5*, *METTL14*, *YTHDF1*, and *YTHDF2*) in PBMCs from patients with RA. The authors showed that METTL3 is upregulated in RA patients compared to healthy controls [110]. YTHDF2 belongs to the family of cytoplasmic reader proteins that alter the degradation and translation of mRNA. It is believed to coordinate LPS-induced inflammatory responses. Yu et al. showed that YTHDF2 knockdown significantly increased the expression of IL-6, TNF-α, IL-1β, and cytokines acknowledged in RA development [121]. Yao et al. assessed the mRNA expression of *YTHDF2* in PBMC from both RA patients and the control group, the association between the mRNA expression of *YTHDF2* and the host inflammatory levels, and the correlation between *YTHDF2* mRNA expression in PBMC and the mRNA of the pro-inflammatory cytokines in these cells [122]. In this study, decreased levels of PBMC mRNA expression of *YTHDF2* were demonstrated in RA patients. Moreover, PBMC *YTHDF2* mRNA expression in RA patients was negatively correlated with erythrocyte sedimentation rate (ESR), CRP levels, and white blood cell (WBC) levels, which are typical markers of inflammation and with serum levels of RF, indicating that YTHDF2 might play a negative regulatory role in RA progression. On the contrary, Wang et al. reported no significant differences in PBMC *METTL14*, *FTO*, *ALKBH5*, *YTHDF1*, and *YTHDF2* mRNA expression between patients with RA and healthy controls [110]. Luo et al. presented lower expression levels of *ALKBH5*, *FTO*, and *YTHDF2* in the peripheral blood of RA patients. However, there were no significant differences in the expression levels of *METTL3*, *METTL14*, and *WTAP* between both groups. Researchers also showed positive correlations between the expression of peripheral blood *FTO* and DAS28-ESR and DAS28-CRP. These findings suggest that *ALKBH5*, *FTO*, and *YTHDF2* share connections with inflammatory processes in RA. The decreased expressions of these modifications in peripheral blood may act as risk factors for RA in the future. The results of this study indicate that the expression of peripheral blood *ALKBH5*, *FTO*, and *YTHDF2* could be a significant step towards identifying novel biomarkers for RA management [106].

## 5. Conclusions and Future Perspectives

To conclude, epigenetics is an important field of study in inflammatory diseases, and current evidence suggests the involvement of DNA and RNA methylation in the pathogenesis of RA. Hypothetically, the upcoming years will uncover novel epigenetic-related therapeutic targets that could indicate the introduction of new treatment strategies. Moreover, as methylation significantly contributes to the profile of expressed genes, the interplay of genetics and epigenetics could demonstrate major pathophysiology mechanisms responsible for disease progression, thus creating opportunities for precision medicine. Monitoring methylation could be used in the diagnosis of RA, which would improve detecting RF- and ACPA-negative RA patients, thus allowing for a more rapid treatment.

## Figures and Tables

**Table 1:** A brief summary of methylation differences observed in RA and their potential involvement in the pathophysiology of the disease.

Gene/CpG Site	Methylation Status	Association with RA	References
CXCR5	Increased methylation	Positively correlated with CRP levels; Suggesting systemic inflammation link.	[76]
HIPK3	Decreased methylation	Negatively correlated with CRP; Predictive potential for seronegative RA patients.	[77]
CDKN2A	Decreased methylation	Lower levels in RA patients compared to controls; Moderate diagnostic power.	[85]
TNF	Decreased methylation	Predictive of RA diagnosis among arthritis subtypes (e.g., psoriatic, reactive arthritis).	[86]
LINE-1	Differential methylation	Associated with MTX response; Possible link to invasive features of synovial cells.	[91]
Multiple CpG Sites	Altered methylation	1511 CpG sites linked to CRP levels; CRP shown to influence methylation.	[78]

**Table 2:** Summary of selected RNA modifications involved in RA.

RNA Modification	Key Genes/Enzymes	Potential Involvement in Rheumatoid Arthritis	References
m6A (N6-methyladenosine)	METTL3, METTL14, WTAP, FTO, ALKBH5, YTHDF1/2/3, YTHDC1/2, hnRNPC, IGF2BP	Regulates inflammation, macrophage proliferation, cytokine production (e.g., IL-6, TNF-α); Influences RNA stability and translation.	[23,103,110]
m5C (Cytosine-5 methylation)	DNMT2, NSUN2, TRDMT1	Reduced m5C in synovial tissues linked to inflammation and RA severity.	[23]
m1A (N1-methyladenosine)	Not specified	Potentially contributes to RA pathogenesis.	[23]

## Data Availability

Not applicable.
